# Learning to sense from events via semantic variational autoencoder

**DOI:** 10.1371/journal.pone.0260701

**Published:** 2021-12-23

**Authors:** Marcos Paulo Silva Gôlo, Rafael Geraldeli Rossi, Ricardo Marcondes Marcacini

**Affiliations:** 1 Institute of Mathematics and Computer Sciences, University of São Paulo, São Carlos, São Paulo, Brazil; 2 Federal University of Mato Grosso do Sul, Três Lagoas, Mato Grosso do Sul, Brazil; Taipei Medical University, TAIWAN

## Abstract

In this paper, we introduce the concept of learning to sense, which aims to emulate a complex characteristic of human reasoning: the ability to monitor and understand a set of interdependent events for decision-making processes. Event datasets are composed of textual data and spatio-temporal features that determine where and when a given phenomenon occurred. In learning to sense, related events are mapped closely to each other in a semantic vector space, thereby identifying that they contain similar contextual meaning. However, learning a semantic vector space that satisfies both textual similarities and spatio-temporal constraints is a crucial challenge for event analysis and sensing. This paper investigates a Semantic Variational Autoencoder (SVAE) to fine-tune pre-trained embeddings according to both textual and spatio-temporal events of the class of interest. Experiments involving more than one hundred sensors show that our SVAE outperforms a competitive one-class classification baseline. Moreover, our proposal provides desirable learning requirements to sense scenarios, such as visualization of the sensor decision function and heat maps with the sensor’s geographic impact.

## Introduction

News portals and social networks publish and disseminate information on the web about various events, such as politics, epidemics, urban violence, finance, climate, natural disasters, among others. Due to the wide variety of useful real-world applications from event analysis, different initiatives aim to identify and store thousands of events daily, such as GDELT (A Global Database of Society: https://www.gdeltproject.org/) and ICEWS (Integrated Crisis Early Warning System: https://dataverse.harvard.edu/dataverse/icews). Furthermore, researchers and data scientists exploit such repositories to support social studies by building indicators from events in different domains [1]. For example, Google Flu Trends [2] is a real-time influenza surveillance system based on aggregated events from web search activities [3]. Radinsky et al. [4, 5] analyzed news events to predict Cuba’s first cholera epidemic in decades and early Arab Spring riots. Yaqub et al. [6] analyzed events extracted from social networks as an alternative to analyzing US elections.

Although event analysis is a multidisciplinary research topic, a common factor among the studies is interpreting web events as a digital social sensor that maps phenomena in our physical world [7, 8]. The pioneering studies for event analysis using machine learning methods are known as Topic Detection and Tracking (TDT) [9, 10] and Event Detection (ED) [11, 12]. A very common definition in these studies is that events are phenomena that occur at a certain time and place, which can currently be extracted from the textual data of the news and social networks [9, 12]. In practice, this definition indicates that neither textual excerpt is an event, but only those we can associate temporal and geographic information to an action reported in the text. In general, events are pre-processed to extract components indicating what happened, when it happened, and where it happened, i.e., events have semantic information associated with spatio-temporal features [13].

Existing methods in both TDT and ED areas rely on text classification to filter and categorize events. However, we argue that event analysis has its own requirements that differentiate it from traditional text classification strategies. For instance, when a human explores events for decision making, he/she implicitly interprets actions represented in the events considering spatio-temporal constraints. Thus, a modern event analysis process should emulate a complex human reasoning: the ability to observe and interpret related events from the past, in locations of interest, to support decision-making processes or make predictions. We call this task *learning to sense* to differentiate it from traditional text classification methods used in TDT and ED areas, i.e., when we want to perform event analysis to build indicators (sensors) that consider events related both by their content and by their spatio-temporal features. In fact, a machine learning method focused on learning to sense must deal with the following challenges:

- Lack of semantics: identifying related events is one of the main challenges of event analysis. Interdependent events may not explicitly have features in common. For example, an event describing the prolonged rainfall shortages in a given region may be related to another event describing a rise in future prices for a given agricultural product. Such events may be related if an agricultural product is produced in that region. Classical models for text representation, such as bag-of-words, fail in event analysis due to their limitation in texts’ semantic representation. A promising alternative is leveraging neural language models to represent event textual data with a higher level of semantics [14, 15]. However, investigating the proper use of such models for learning to sense is an underexplored challenge.
- Sensor maps: although event analysis tasks mainly involve predictive classification models, these tasks still depend on exploratory and descriptive analysis. Users are generally interested in the same functionality expected in a classic sensor [12, 16, 17], i.e., a sensor map that visually indicates which events belong to the interest class. However, classification methods usually represent events in higher dimensions, which makes the direct visualization of sensor maps unfeasible. An alternative is to use dimensionality reduction techniques in the event data, such as t-SNE and PCA. Still, these techniques require the reconstruction of the projections for each new instance [18, 19]. Thus, integrating the generation of 2D sensor maps within the event classifier is a desirable property for event sensing.
- Spatio-temporal features: geographical and temporal information is associated with events, in addition to textual information. However, existing methods use time and location information only as an event filter [12, 15]. We argue that an adequate event representation for learning to sense should preserve both spatio-temporal and textual features in a low dimensional latent space since geographical relationships and trends are important factors in determining whether events are related.

Recently, some studies for event analysis have been proposed to overcome the above drawbacks. Event2Vec [14] explores representation learning from graphs generated by the events’ textual information. TED (Temporal Event-Driven) was proposed by [20] to learn temporal embeddings and compare textual information between events over time. Newsmap [21] combines named entity extraction and text classification for the geographical focus of event news stories. Although prior studies are promising for event analysis tasks, their application is limited to some event information component (temporal or geographical). Learning to sense tasks requires a representation model that considers both textual, temporal, and geographical information. Thus, we raise the following question: how learn a low-dimensional representation (2D sensor maps) for events while preserving semantic similarity and spatio-temporal data?

This paper introduces a method for learning to sense that addresses the challenges discussed above. We present a Semantic Variational Autoencoder (SVAE) method for learning to sense tasks. First, our method explores the state-of-the-art BERT (Bidirectional Encoder Representations from Transformers) neural language model [22, 23] to learn general-purpose semantic and spatio-temporal features from the events. Second, given a set of events (class of interest) for training a sensor, we use event semantic features as input to a Variational Autoencoder. In this case, we want to map the BERT semantic space into a two-dimensional sensor map, where events of the interest class are allocated close to each other to form high-density regions. Moreover, our SVAE preserves the semantic and spatio-temporal features that are implicit in the training set by using the bottleneck layer of the Variational Autoencoder as final event features, which facilitates the sensor learning using one-class classification methods. Unlike the existing methods that require a prior definition of the geographic or temporal information, our SVAE can implicitly embed this information into the event representation model when it is important to identify new events of the interest class.

We carried out a thorough experimental evaluation involving 183 collections of real-world events extracted from the GDELT project. In addition, we compared our proposal with a competitive baseline using Embedding-based One-Class SVM from BERT event embeddings. The experimental results reveal that using the proposed SVAE leads to learning sensors with higher *F*_1_ values in 177 of 183 event collections, even using a low-dimensional event representation model. Also, our model naturally enables exploratory and visual analysis of events from 2D sensor maps.

## Preliminaries

### One-class learning

Let the domain of instances be X, Y be the domain of labels, and let a training sample $(x_{i},y_{i}{)}_{i=1}^{m}$, in which $x_{i}\in R^{n}$ is the feature vector of the *i*-th example, and *y*_*i*_ is a label associated with the example **x**_*i*_. The goal of one-class learning (OCL) is to learn a function f:X→Y, in a way that *f*(**x**) predicts the label *y* correctly on future data, and *y* ∈ {+ 1, −1}, i.e., if the new example belongs to the positive (interest) class, or not [24]. However, different from a binary learning problem, the training examples’ labels belong only to the positive class, i.e., *y*_*i*_ = +1, ∀1 ≤ *i* ≤ *m*.

During decades, one-class learning was performed considering basically generative, boundary, or reconstruction methods, such as One-Class Support Vector Machines, Parzen Density Estimation, and proximity-based methods such as *k*-Means or *k*-Nearest Neighbors [24–26]. Usually, those methods require substantial feature engineering to be effective in several tasks, mainly those involving text data [27, 28]. On the other hand, deep learning techniques allow obtaining relevant features automatically [18, 29]. Also, the quality of the representations learned by deep learning techniques or the direct application to perform classification can surpass the results of other approaches [18, 27].

### Deep one-class learning

Due to the advances in deep learning, the proposals and use of deep learning-based approaches to perform one-class classifications increased notably in the last years [29–32]. We refer to those approaches as deep one-class learning (DOCL). The use of DOCL can be observed in different areas such as natural language processing [33, 34], computer vision [31], and recommendation systems [30], to cite a few.

**Fig 1:**
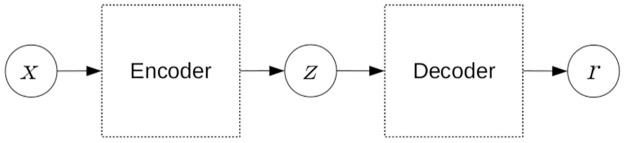
Basic structure of an autoencoder.

DOCL can be used directly to perform predictions [35, 36] or to learn in an unsupervised way a low-dimensional representation, or a latent space, which will feed other one-class learning algorithms [31, 33]. The DOCL is mostly implemented through autoencoder architecture, which is composed of two steps: encoder and decoder. The encoder and decoder can be viewed as two functions: *z* = *f*(*x*) and *r* = *g*(*z*), in which *f*(*x*) maps the original example *x* to a low-dimensional space (encoding), and *g*(*z*) performs the reconstruction of *x* by mapping *z* to the original space (decoding) [37]. The basic structure of an autoencoder is presented in Fig 1.

Given a training set $X=\{x_{i}|x_{i}\in R^{n}\}$, with 1 ≤ *i* ≤ *m*, *n* as the number of dimensions in the original space, and assuming that encoder and decoder functions are implemented by neural networks, the mapping steps of an autoencoder can be formally defined by:

$$\begin{array}{c} autoencoder=\{\begin{array}{c} z=f(W_{e},B_{e};x)\\ r=g(W_{h},B_{h};z) \end{array} \end{array}$$ (1)

in which **W**_*e*_ and **B**_*e*_ are respectively the weights and biases of the encoding neural network, and **W**_*h*_ and **B**_*h*_ are weights and biases of the decoding neural network.

The neural network parameters are learning in a way to optimize the following function [37]:

$$\begin{array}{c} J\left(\Theta \right)=\frac{1}{n}\sum_{i=1}^{n}||x_{i}-r_{1}|{|}_{2}^{2} \end{array}$$ (2)

in which Θ = (**W**_*e*_, **B**_*e*_;**W**_*h*_, **B**_*h*_). Thus, the goal of an autoencoder is to produce an output similar to the input. Since the hidden layers usually consist of some neurons much lower than the input (e.g., bottleneck layer), the reconstructions are only possible if the hidden layers’ weights capture the most representative features of the input data [32]. Also, the low dimension representation learned by autoencoders can be less computational expensive and presents a higher quality for several tasks in comparison with other traditional approaches such as Principal Component Analysis (PCA), Isometric Feature Mapping (Isomap), Locally Linear Embedding (LLE), or Stochastic Neighbor Embedding (SNE) [18].

A classic autoencoder is a feedforward fully-connected neural network with *o* layers, in which the *i*-th and (*o* − *i*)-th layers have the same number of neurons [36]. However, due to the advanced neural network architectures and their useful characteristics for some domain applications, different neural network architectures have been used in an autoencoder’s encoding and decoding steps. In literature, we can observe that convolutional, recurrent, and variational autoencoders are commonly used for text data [18, 33, 34, 38–40].

### Variational autoencoder for text

A Variational Autoencoder (VAE) is also based on the regularization function of an autoencoder (Eq 1). Still, the goal is to estimate the probability density function of the training data [39]. Thus, VAEs are a generative model. VAEs are used in different data types, such as images and sounds, but attracted attention to text data since generative models are used in several NLP tasks [38].

One of the main characteristics of VAE is that instead of forwarding the latent values inferred by the encoder to the decoder directly, VAEs use them to calculate the parameters of a distribution model, e.g., mean and a standard deviation in case of a normal distribution. Also, the input to the decoder is a sample generated through the inferred parameters of the distribution. Thus, VAEs can generate new instances of the data [41].

Given a variable *x* and assuming that a continuous latent variable *z* generates *x*, a VAE assumes a probability function:

$$\begin{array}{c} p\left(z|x\right)=\frac{p(x|z)p(z)}{p(x)} \end{array}$$ (3)

in which,

$$\begin{array}{c} p(x)=\int p(x|z)p(z)dz \end{array}$$ (4)

Since integrals are intractable, VAE aims to optimize the marginal log-likelihood *p*(*x*) = ∫*p*_Θ_(*z*)*p*(*x*|*z*)*dz* that can be written as [38]:

$$\begin{array}{c} \text{log}p_{\Theta }\left(x\right)=KL(q_{\Phi }\left(z|x\right)||p_{\Phi }\left(z|x\right))+L\left(\Theta ,\Phi ;x\right) \end{array}$$ (5)

where

$$\begin{array}{c} L\left(\Theta ,\Phi ;x\right)=-KL(q_{\Phi }\left(z|x\right)||p_{\Phi }\left(z\right))+E_{q_{\Phi }\left(z|x\right)}\text{log}\;p_{\Theta }\left(x|z\right) \end{array}$$

and KL is Kullback–Leibler divergence, *q*_Φ_(*z*|*x*) is the variational approximation to the posterior *p*(*z*|*x*) (or the distribution of the encoded variable given the decoded one), *p*(*z*) is the prior knowledge, e.g., a multivariate Gaussian N(z;0,1), $E_{q_{\Phi }\left(z|x\right)}$ is an approximation of *z* through a reparameterization trick and differentiate through the sampling stage, and *p*_Θ_ is the distribution of the decoded variable given the encoded one. In [38] is presented the impact of different priors *p*(*z*) in language and document modeling. While [38] uses VAEs directly in the token sequence extracted from the texts, our approach refines the textual embedding representation from a previous semantic space obtained by a BERT neural language model, thereby taking advantage of the pre-training and existing general-purpose knowledge of the BERT model.

## Learning to sense from events

**Fig 2:**
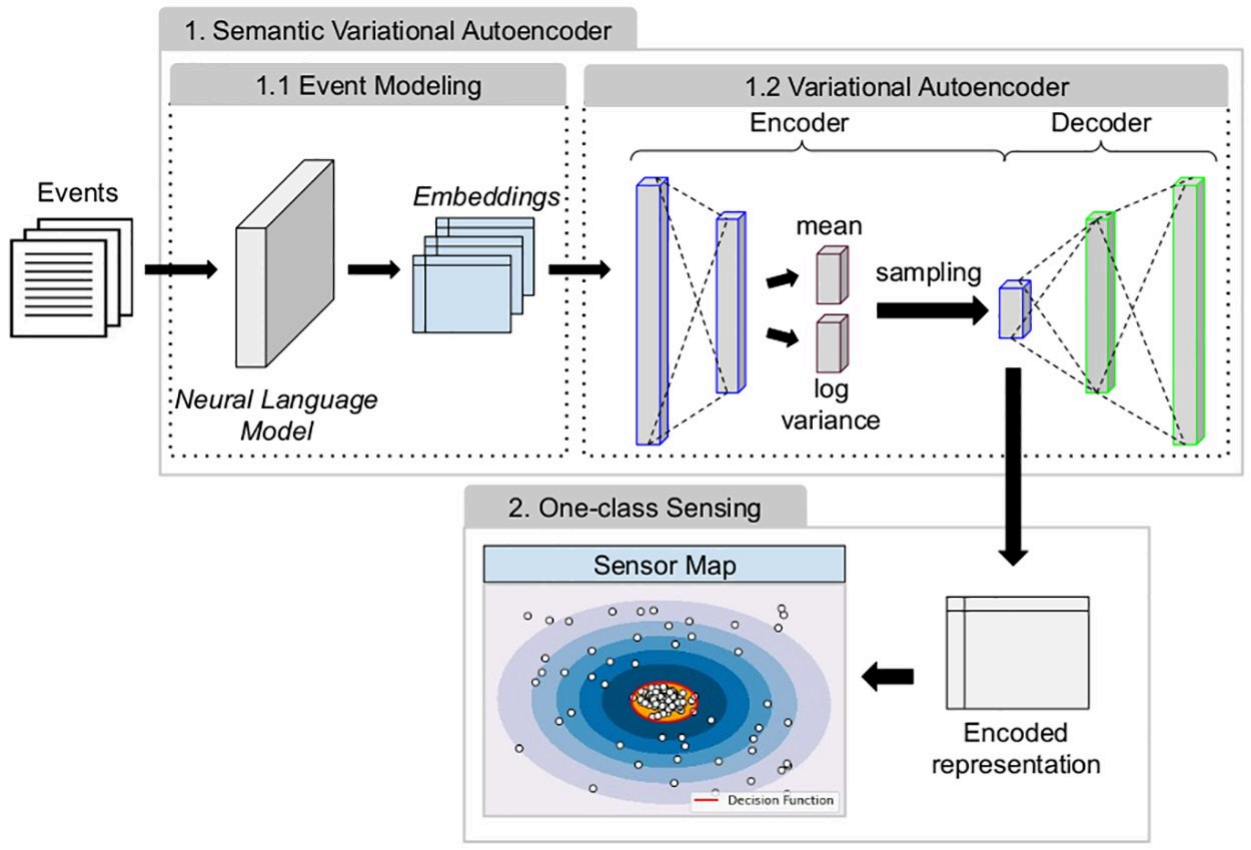
An overview of the proposed learning to sense method.

The proposed learning to sense method is based on two steps. In the first step, we represent the event dataset using a general-purpose neural language model to capture semantic relationships from the textual event information via contextual word embeddings (event modeling). We use contextual word embeddings trained in large news datasets to preserve both general topics and expressions related to locations (city and country names) and temporal expressions. For example, such embeddings can capture relationships between cities and state capitals and words related to time periods (e.g., Christmas and December). In the second step, we use a Variational Autoencoder to extract non-linear features from semantic embeddings and generate a low-dimensional sensor map from a set of training events. Our sensor map is a 2D event space that preserves the original embeddings’ local structures, such as semantic similarities and spatio-temporal relations. In our proposal, we defined a Semantic Variational Autoencoder (SVAE) using a VAE to extract a low-dimensional event representation from BERT language models, guided by the reconstruction (via encoder-decoder structure) of the events of the interest class. Unlike dimensionality reduction techniques (e.g., t-SNE and PCA), a trained SVAE can generate representations of new events, i.e., the encoder step is used as a predictive function to allocate the new events to a sensor map region. Any one-class classifier can be used for the sensing step from the sensor map. In our proposal, we use the One-Class SVM. An overview of the proposed method is presented in Fig 2. In the next sections, we present details of each step.

### Event modeling

We present the event modeling task as the probability of the event *e*_*i*_ to be related to a set of events {*e*_1_, *e*_2_, …, *e*_*i*−1_}. The concept of related events can be interpreted as events of the same class, i.e., we aim to identify the occurrence of events about a certain phenomenon of interest. In general, this task has been formulated in the context of neural networks through the conditional probability of Eq 6, where *θ* represents model parameters and *h*_*i*_ = *f*(*h*_*i*−1_, *e*_*i*_, *θ*) represents a hidden state vector that aims to sequentially encode known events, e.g., using temporal information from events.

$$\begin{array}{c} p\left(e_{i}|\left\{e_{1},e_{2},...,e_{i-1}\right\}\right)=p\left(e_{i}|h_{i-1},\theta \right) \end{array}$$ (6)

A simple strategy to address this modeling is to consider that events are composed of a sequence of words, and a concatenation of all word sequences forms the dataset. We can obtain an approximation of Eq 6 via the traditional *n*-grams model, i.e., computing conditional probabilities tables for each word given a subset of preceding words. In fact, this is a classic count-based language model strategy and has well-known limitations, such as the curse of dimensionality and difficulty in dealing with large sequences of *n*-grams. Neural language models have been explored recently to overcome such limitations, especially with the use of LSTM for learning long-term dependencies between word sequences of events.

We explore leveraging pre-trained neural language models for event modeling. In particular, we use the state-of-the-art BERT (Bidirectional Encoder Representations from Transformers) for the encoded representation of events (event embedding space). BERT is a contextual neural language model, where the representation of a word is a function of the entire text in which it occurs. The model is pre-trained in a large textual corpus so that contextual representations can encode general semantic relations that are useful for event analysis, such as geographical relations between words (e.g., countries and capitals), temporal relations (e.g., dates and expressions), people’s names, and their relations with organizations and governments, as well as other types of named entities. Furthermore, BERT is trained in the task of predicting the next sentence, which is very useful for identifying relationships between texts from two events.

In the context of event modeling, given the word sequence of the event *e* = (*w*_1_, *w*_2_, …, *w*_*T*_), BERT model first generates a corrupted $\hat{e}$ version of the event, where approximately 15% of the words are randomly selected to be replaced by a special token called [MASK]. Thus, the objective function is to reconstruct the masked tokens $\bar{e}$ from $\hat{e}$, according to Eq 7,

$$\begin{array}{c} \underset{\theta }{\text{max}}\;log\;p\left(\bar{e}|\hat{e},\theta \right)\approx \sum_{t=1}^{T}m_{t}log\;(\frac{\text{exp}(h_{\theta }{\left(\hat{e}\right)}_{t}^{⊤}{\vec{w}}_{t})}{\sum_{w^{\prime }}\text{exp}\left(h_{\theta }{\left(\hat{e}\right)}_{t}^{⊤}\vec{w^{\prime }}\right)}) \end{array}$$ (7)

where $\vec{w}$ indicates the embedding of *w*; the $h_{\theta }{\left(\hat{e}\right)}_{t}$ is a sequence of *T* hidden state vectors according to parameters *θ* from the neural network model (e.g. Transformers); and *m*_*t*_ = 1 indicates when *w*_*t*_ is masked.

In [22] are provided pre-trained general-purpose BERT models. We use a fine-tuned version of the BERT model for the task of similarity sentences from a multilingual news corpus, as discussed in [23]. In this case, the task consists of adjusting the sentence embeddings to maximize the cosine similarity of sentence pairs. Therefore, each sentence shares several event components, such as general tokens, geographic information, and time expressions.

### Variational autoencoder

The general purpose embeddings obtained in the previous step are processed in this step, Variational Autoencoder, considering a set of events of the interest class. Two assumptions are considered during Variational Autoencoder training for learning to sense: (1) events must be mapped to a two-dimensional space that represents a sensor map; and (2) the distance of the events on the sensor map must reflect the textual similarity, as well as the temporal and geographical information of the class of interest. Each assumption is described in detail below.

**Assumption 1** *An event sensor is a two-dimensional map where events of interest are positioned close to each other to generate high-density regions. In contrast, events of non-interest are positioned far from these regions*.

We use two-dimensional maps to represent the event sensors for two reasons. First, we maintain an analogy with human experts’ classic sensor maps for analytical intelligence tasks and decision support systems. Second, event analysis lacks explainable models during decision-making processes, and two-dimensional maps allow the visualization of decision functions that indicate how close new events have been allocated to the class of interest and explore other nearby events. Obviously, according to the application requirements, we can easily increase the event sensors’ dimensional space. However, two-dimensional maps were effective in the experiments carried out in this work and satisfying the properties of visualization and explainability.

We propose a Semantic Variational Autoencoder (SVAE). We chose VAE because it is a powerful method for non-linear feature extraction (or dimensionality reduction). Also, VAEs generalize an AE by not only learning a representation but also learning how to generate new instances of the data. This generative characteristic is obtained through the imposition of a specific structure on the hidden layer: the activation in the hidden units should be drawn from the standard Gaussian with zero mean and unit variance [38]. By the end, it is noteworthy that these characteristics make VAEs stand out in tasks related to textual data such as language modeling [38, 39, 42], document modeling [38, 43], imputing missing words [39], semisupervised classification [44, 45], text representation for clustering [33], text representation for classification and conditional sentence generation [42].

Our SVAE is semantic mainly because we use fine-tuned contextual BERT embeddings of events as input data. The proposed VAE method has an encoder $q_{\phi }\left(z|\vec{e}\right)$ to map event embeddings $\vec{e}$ in a two-dimensional vector space *z* = [*z*_(*x*)_, *z*_(*y*)_]^⊤^, and a decoder $p_{\theta }\left(\vec{e}|z\right)$ that uses latent variables *z* to reconstruct the original embedding. Parameters *ϕ* and *θ* are learned from the neural network for encoding and decoding, respectively.

$$\begin{array}{c} -E_{q_{\phi }\left(z|\vec{e}\right)}\left(\text{log}\;p_{\theta }\left(\vec{e}|z\right)\right)+KL\left(q_{\phi }\left(z|\vec{e}\right)\|p\left(z\right)\right) \end{array}$$ (8)

Eq 8 is the loss minimization function of the VAE, where the first term consists of a loss reconstruction, and the second term consists of the Kullback-Leibler (KL) divergence between the learned two-dimensional space *z* and a prior distribution *p*(*z*). Note that KL measures the difference between two probability distributions. In our VAE, p(z)=N(z;0,I) with mean of zero, where **I** is the identity matrix; and $q_{\phi }\left(z|\vec{e}\right)=N\left(z;u_{z},\sum_{z}\right)$ is the normal distribution with mean of $u_{z}={\left[u_{z_{\left(x\right)}},u_{z_{\left(y\right)}}\right]}^{⊤}$ and the covariance matrix ∑_*z*_ for the latent variables sampling.

We train the SVAE from a set of interest events *E* = {*e*_1_, *e*_2_, …, *e*_*n*_}, and we use the trained encoder to generate the relation $\left(\vec{e},z\right)$ between the event BERT embedding $\vec{e}$ and its respective coordinates *z* = (*z*_(*x*)_, *z*_(*y*)_) on the sensor map. The compression strategy of the SVAE encoder tends to extract features to map events of the class of interest in the same region in two-dimensional space, thereby generating regions of high-density. On the other hand, events with different features (BERT embeddings) concerning the class of interest tend to be mapped in more distant regions.

**Assumption 2** *Distances* *d*_*sensor*_(*e*_*i*_, *e*_*j*_) *on the two-dimensional map maintain proportionality between textual content proximity* *d*_*text*_(*e*_*i*_, *e*_*j*_) *and spatio-temporal proximity* *d*_*geotime*_(*e*_*i*_, *e*_*j*_) *between events*, *i.e*., *d*_*sensor*_(*e*_*i*_, *e*_*j*_) ∝ *d*_*text*_(*e*_*i*_, *e*_*j*_) ∝ *d*_*geotime*_(*e*_*i*_, *e*_*j*_).

Distances between events on the sensor map must preserve local structures of the event embeddings space. In other words, we want to maintain a level of proportionality between spatial relationships in the sensor map with the semantic relationships identified in the neural language model. In fact, the proportionality relationship *d*_*sensor*_(*e*_*i*_, *e*_*j*_) ∝ *d*_*text*_(*e*_*i*_, *e*_*j*_) between sensor map and BERT embeddings, respectively, is obtained straightforwardly from the VAE training. Here, we discuss the proportionality relationships with spatio-temporal features of the events.

We argue that there are two main strategies to preserve spatio-temporal features of events in the sensor map. The first strategy is to incorporate spatio-temporal constraints during VAE training, in which the minimization function would be penalized when violating such constraints. However, we adopted a second strategy because it is simpler and more intuitive, which assumes that pre-trained neural language models already consider spatio-temporal features in their contextual word embeddings. This assumption is strengthened when we analyze recent studies that explore pre-trained neural language models for NER tasks [22]. Also, suppose the class of interest contains events associated with certain locations and events over certain time periods. In that case, VAE will preserve these features during your training due to the bottleneck strategy of the encoder-decoder structure.

### One-class sensing

Now, consider the set of interest events *E* = {*e*_1_, *e*_2_, …, *e*_*n*_} represented on the sensor (two-dimensional) map feature space, i.e., *E*_(*z*)_ = {*z*_1_, *z*_2_, …, *z*_*n*_}. Our goal is to find a decision function capable of encompassing such events in the sensor map, but also capable of identifying possible outliers in the training set. We use the One-Class Support Vector Machine formulation proposed by [46] to learn such a decision function.

A naive strategy to solve one-class sensing is to find the hypersphere of minimum volume enclosing the event features vectors, according to Eq 9,

$$\begin{array}{c} \mu_{\left(c\right)}=\underset{\mu \in H}{\text{arg min}}\underset{1\leq i\leq n}{\text{max}}{\left\|\upsilon \left(z_{i}\right)-\mu \right\|}^{2} \end{array}$$ (9)

where *H* is the feature space associated with an SVM kernel function *φ*, and *μ*_(*c*)_ is the center of the hypersphere in which the largest distance between events *φ*(*z*) to *μ*_(*c*)_ is minimal. The main drawback of this solution is that outlier events can arbitrarily increase the hypersphere’s volume, and any new events can be classified as belonging to the class of interest.

To minimize the effect of outlier events, we can add a regularizer to make the hypersphere more flexible and accept a certain margin violation level. In this case, each *φ*(*z*_*i*_) feature vector is associated with a *ε*_*i*_ parameter that measures the external distance between *φ*(*z*_*i*_) and the hypersphere surface. We also use a parameter *ν* ∈ (0, 1] to define the hypersphere volume’s softness level, where the closer to 1, the greater the softness level.

$$\begin{array}{c} \begin{array}{cc} \underset{\mu ,\varphi ,r}{\text{min}}\; & r^{2}+\frac{1}{n}\sum_{i=1}^{n}\frac{\varepsilon_{i}}{\nu } \end{array} \end{array}$$ (10)

subject to

$$\begin{array}{c} \begin{array}{cc} \; & \|\varphi \left(z_{i}\right){-\mu \|}^{2}\leq r^{2}+\varepsilon_{i}\;\;\;\forall i=1,...,n \end{array} \end{array}$$ (11)

Eq 10 formalizes this solution’s minimization function, where we now want to minimize both the hypersphere squared radius *r* and the number of margin violations. The parameter *ν* configures this trade-off, allowing generalizing the one-class sensing by enclosing most events in the dense regions of the sensor map and allowing to ignore possible outlier events, as illustrated in the sensor map decision function in Fig 2.

## Experimental evaluation

### Event collections

We used 183 event collections from the GDELT project database, which monitors real-time events worldwide. Each event collection represents a topic chosen by the International Press Telecommunications Council (IPTC) taxonomy. First, we generate a CSV file for all topics from IPTC (1203 topics). Thus, we built the event collection by checking if the event contained the topic in its title. We collected the events with more than 4000 events, generating a total of 183 collections. We defined 6000 events as the maximum limit for each collection.

We determine an event class as a set of events related to a topic, i.e., an event collection. Then, we use the google cloud big query (https://cloud.google.com/bigquery) to access the GDELT tables that contained the events to collect them. Because of limits with access, we use the *gdelt-bq.gdeltv2.gkg_partitioned* table that is partitioned by time. For instance, if a topic is agriculture, we made the query represented by Algorithm 1 that collect events related to agriculture in September 2019. If this query does not return 6000 events, we use the next month until complete the collection.

**Algorithm 1**. Query to collect events related to the agriculture theme in September 2019.

SELECT

 Min(GKGRECORDID) Id,

 Min(DATE(_PARTITIONTIME)) Date,

 REGEXP_EXTRACT(Extras, ‘<PAGE_TITLE> (.*) </PAGE_TITLE>’) Title

FROM

 *‘gdelt–bq.gdeltv2.gkg_partitioned’*

WHERE

 EXTRACT(MONTH FROM DATE(_PARTITIONTIME)) = 9 and

 EXTRACT(YEAR FROM DATE(_PARTITIONTIME)) = 2019 and

 REGEXP_CONTAINS(Extras, ‘.*TITLE.*’) and

 REGEXP_CONTAINS(REGEXP_EXTRACT(Extras, ‘<PAGE_TITLE> (.*) </PAGE_TITLE>’), ‘agriculture’)

GROUP BY Title

ORDER BY Date ASC

LIMIT 6000

The 183 event datasets are:

- Restaurant—Opera—Employee—Logistics—Imports—Flood—Tariff
- Metal—Ethics—War—Investments—School—Revolution—Society—People
- Marathon—Adults—Theft—University—Gender—Assault—Traffic—Retail
- Massacre—Water—Rivers—Lifestyle—Welfare—Media—Impeachment—Illness
- Fraud—Teachers—Pope—Fashion—Fire—Terrorism—Environment—Currency
- Social media—Boxing—Architecture—Birthday—Theatre—Fiction—Drought
- Mosque—Police—Disaster—Sailing—Tobacco—Transfer—Education—Cancer
- Diet—Pension -Cinema—Advertising—Pandemic—Retirement—Radio—Judge
- Animal—Coal—Surveillance—Ceremony—Management—Loans—Suicide
- Arson—Trend—Immigration—Labour—Automotive—Festival—Prison—Epidemic
- Petrol—Language—Party—Prices—Parliament—Bullying—Poverty—Economy
- Crime—Vaccines—Health—Recession—Employment—Music—College—Wildfire
- Abortion—Halloween—Bribery—Securities—Software—LGBTQ—Government
- Mining—Transport—Discrimination—Divorce—Toy—Agriculture—Defence -
- Christmas—Family—Culture—Racism—Surgery—Privacy—Adoption—Medicine
- Rugby—Unemployment—Parks—Voting—Bar—Constitution—Unions—Design
- Insurance—Anniversary—Sport—Game—Homicide—Bankruptcy—Inflation
- Volleyball—Hurricane—Regulations—Plant—Earthquake—Painting—Consumers
- Mortgage—Tourism—Election—Witness—Farms—Earnings—Hunting—Holiday
- Medicaid—Beverage—Shareholder—Grocery—Musical—Fishing—Wedding
- Court—Children—Series—Justice—Therapy—Medicare—Stocks—Students
- Television—Politics—Arrest—Charity—Newspaper—Democracy—Golf
- Investigation—Exports—Banking—Weather—Dance—Celebrity Hospital
- Kidnapping—Marriage—Church—Law—Easter—Nature—Cafe—Bonds—Drama

### Experimental setup

Our work used the pre-trained neural language model proposed in [23] for event modeling via contextual word embeddings. In this model, each event text is represented in a vector of 512 features.

We carried out tests in ten event collections to choose the parameters of our SVAE, such as the number of layers and neurons in architecture ({512, 256, 128, 64, 2, 64, 128, 256, 512}, {512, 256, 64, 2, 64, 256, 512} and {512, 64, 2, 64, 512}), epochs ({1, 2, 3, 4, 5, 6, 7, 8, 9, 10, 25, 50, 100}), learning rate ({0.001, 0.01}), optimization algorithm ({*Adam*, *RMSprop*}), activation function ({*tanh*, *relu*, *linear*, *sigmoid*}) and batch size ({32, 64}). First, we separate the events into a train set (only events of interest) and a test set (events of interest and non-interest). Second, we visually analyzed how the test examples were behaving when represented by SVAE. Therefore, we chose the parameters in which SVAE generated better representations (test events of interest closer to train events and non-interest events further away from train events). We present the chosen parameters below.

**Fig 3:**
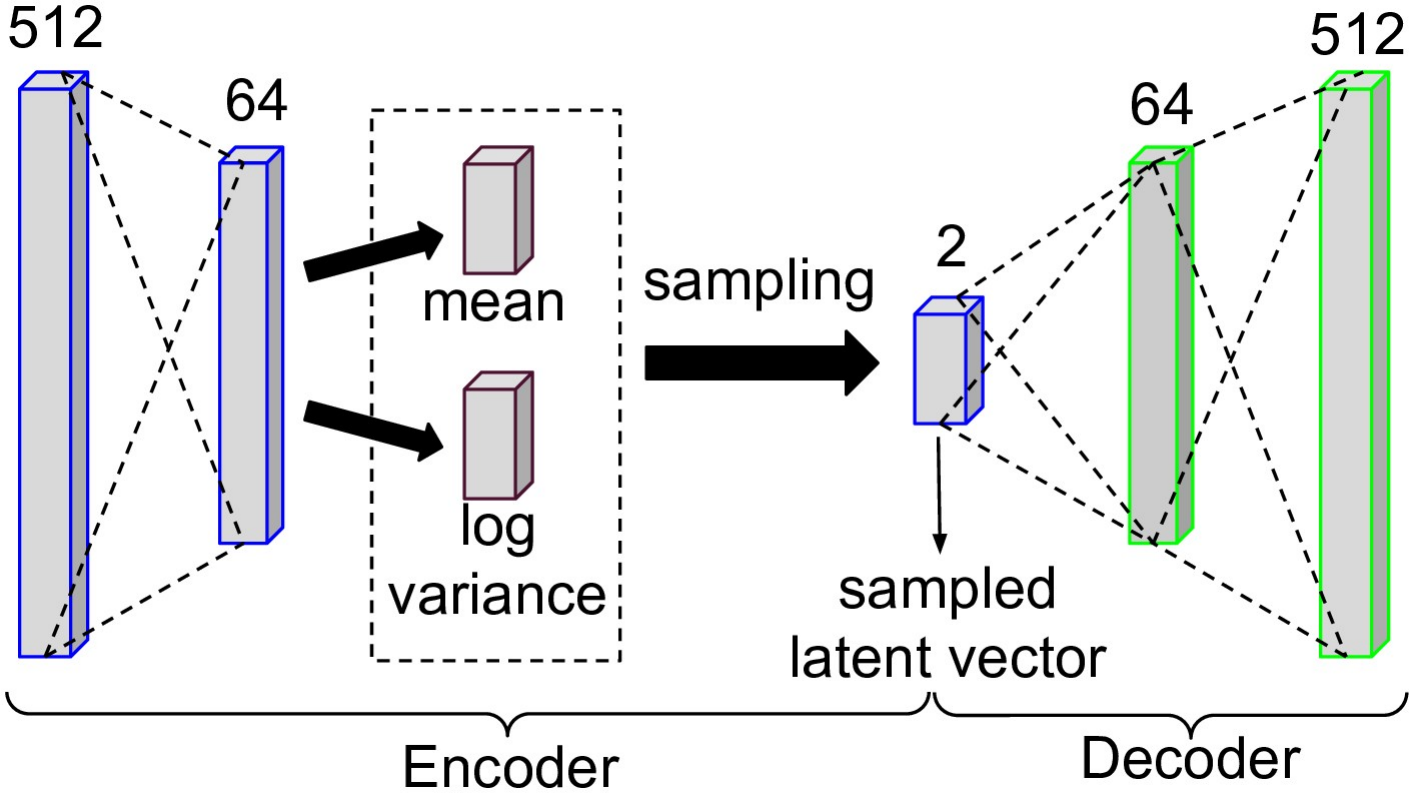
Variational autoencoder architecture.

Fig 3 shows our SVAE architecture used in the experimental evaluation. The values above each layer of Variational Autoencoder are the number of neurons used in each layer. The encoder has two dense layers, one with 512, one with 64, respectively. The bottleneck has size 2. The decoder also has two dense layers, the first with 64, and the second with 512. In the SVAE training step, we used the maximum number of epochs = {1, 2, 3, 4, 5, 6, 7, 8, 9, 10}, the learning rate = 0, 001, optimization algorithm = Adam, activation functions = {*relu*, *sigmoid*} and batch_size = 64.

We used the One-Class SVM (OCSVM) algorithm to perform the sensor learning from the sensor map learned by the SVAE. We choose the RBF kernel, with generally outperforms considering other kernels such as the linear, polynomial or sigmoid [47], and use the *ν* parameter equals 0.01 * *q*, *q* ∈ [1‥^9^] and 0.05 * *q*, *q* ∈ [2‥^19^]. As a baseline for comparison, we used OCSVM directly from contextual word embeddings. We call this strategy Embedding-based OCSVM (EOC-SVM), and it represents a competitive baseline since it already considers the semantic and spatio-temporal features from the neural language model. It is noteworthy that the EOC-SVM input is the same as the SVAE input. Our SVAE proposal, on the other hand, generates a two-dimensional sensor map from the embeddings that aims to extract non-linear features and preserve the semantic and spatio-temporal features of the interest events. Thus, we train the OCSVM from the events of the sensor map.

We used 1/3 of the collection with the oldest dates for the training stage and the other 2/3 in the test stage for each event collection. For evaluation purposes, we randomly add events from other collections to build the test set of size equal to the class of interest test events. We use the same random events to construct the test set for EOC-SVM and SVAE. Furthermore, we generate the results through an average of 10 runs. Each run has a seed to collect random events from other collections. The classification performances were analyzed using the metrics of precision, recall, and *F*_1_-Score [24]. The equations of *F*_1_-Score, Precision (P) and Recall (R) are given respectively by:

$$\begin{array}{c} F_{1}=\frac{2\cdot P\cdot R}{P+R}, \end{array}$$ (12)

$$\begin{array}{c} P=\frac{TP}{TP+FP}, \end{array}$$ (13)

$$\begin{array}{c} R=\frac{TP}{TP+FN}, \end{array}$$ (14)

in which *TP* (True Positives) refers to the number of correctly classified events of the interest class; *FP* (False Positives) refers to the number of wrongly classified events as belonging to the interest class; and *FN* (False Negatives) refers to the number of wrongly classified events as belonging to the non-interest class.

A repository (https://github.com/GoloMarcos/SVAE-plos-one) with the source code of the algorithms, the textual representations, and the most detailed results are provided for reproducibility purposes.

### Results and discussion

**Fig 4:**
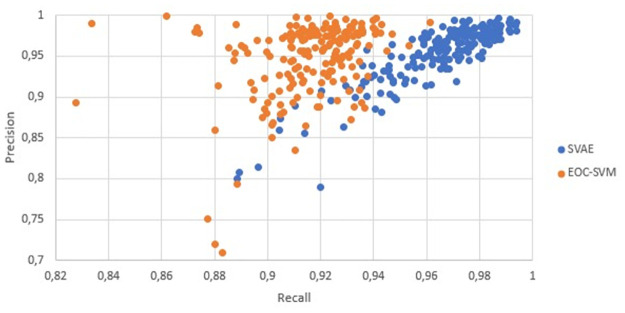
General comparison of precision and recall between the proposed SVAE (blue) and the EOC-SVM baseline (orange) over the 183 event collections.

Fig 4 presents a general comparison between the proposed SVAE and the EOC-SVM through a precision/recall graph. Each point on the graph represents an event collection. The orange points correspond to the classification performance on event collections obtained by EOC-SVM, and the blue points are obtained by our SVAE. We note that SVAE and EOC-SVM methods obtained similar precision values. However, SVAE presented higher recall values in comparison with EOC-SVM. Thus, when considering the *F*_1_-Score, which is the harmonic average between precision and recall, SVAE will have higher *F*_1_-Score values in comparison with EOC-SVM.

Table 1 presents the results of the *F*_1_-Score obtained by SVAE and EOC-SVM. Due to space constraints, we present a comparison with 172 event collections. The left part shows the 86 event collections in which the proposed SVAE obtains the greatest *F*_1_-Score differences compared to EOC-SVM. The right part is analogous but shows the 86 event collections in which the EOC-SVM obtains its best results compared to SVAE. Also, we underline the collection that presents statistically significant differences in an ANOVA test. We observed that EOC-SVM obtains higher *F*_1_-Score values than SVAE only in six event collections. Consequently, SVAE has better performance in 96.7% of event collections. Moreover, when SVAE presents better results than EOC-SVM, the differences are much higher than when EOC-SVM presents better results than SVAE.

**Table 1:** *F*_1_-Score values for 172 event collections to compare SVAE and EOC-SVM sensor learning methods.

Top 86 collections with best results for SVAE				Top 86 collections with best results for EOC-SVM			
Event Collection	SVAE	EOC-SVM	Difference	Event Collection	SVAE	EOC-SVM	Difference
Society	0.8840	0.7916	**0.0924**	Bribery	0.8497	0.8694	-0.0197
Shareholder	0.9609	0.8790	**0.0819**	War	0.8533	0.8706	-0.0173
Boxing	0.9694	0.8938	**0.0756**	Arson	0.9140	0.9241	-0.0101
Wildfire	0.9755	0.9048	**0.0707**	Earnings	0.9314	0.9387	-0.0073
Management	0.9676	0.9011	**0.0665**	Tariff	0.8887	0.8897	-0.0010
Rugby	0.9865	0.9253	**0.0612**	Newspaper	0.9379	0.9387	-0.0008
People	0.8459	0.7865	**0.0594**	Kidnapping	0.8948	0.8933	0.0015
Golf	0.9815	0.9258	0.0557	Arrest	0.9125	0.9109	0.0016
Metal	0.9785	0.9229	0.0556	Theft	0.9242	0.9216	0.0026
Assault	0.9113	0.8592	0.0521	Grocery	0.9481	0.9437	0.0044
Design	0.9704	0.9223	0.0481	Election	0.9629	0.9582	0.0047
Series	0.9322	0.8865	0.0457	Fraud	0.9366	0.9315	0.0051
Marathon	0.9686	0.9233	0.0453	Impeachment	0.9569	0.9491	0.0078
Language	0.9776	0.9338	0.0438	Charity	0.9385	0.9293	0.0092
Retirement	0.9777	0.9339	0.0438	Traffic	0.9331	0.9238	0.0093
Bonds	0.9352	0.8918	0.0434	Recession	0.9483	0.9388	0.0095
Nature	0.9188	0.8756	0.0432	Cinema	0.9615	0.9512	0.0103
Adults	0.9679	0.9248	0.0431	Bullying	0.9542	0.9436	0.0106
Parks	0.9771	0.9344	0.0427	Diet	0.9681	0.9565	0.0116
Television	0.9677	0.9250	0.0427	Coal	0.9746	0.9620	0.0126
Parliament	0.9640	0.9214	0.0426	Crime	0.9220	0.9093	0.0127
Software	0.9845	0.9422	0.0423	Birthday	0.9715	0.9587	0.0128
Regulations	0.9614	0.9193	0.0421	Retail	0.9231	0.9099	0.0132
Witness	0.8804	0.8384	0.0420	Judge	0.9449	0.9311	0.0138
Revolution	0.9680	0.9261	0.0419	Investigation	0.9193	0.9052	0.0141
Lifestyle	0.9376	0.8958	0.0418	Law	0.8996	0.8852	0.0144
Investments	0.9797	0.9380	0.0417	Social-media	0.9550	0.9393	0.0157
Opera	0.9612	0.9196	0.0416	Hospital	0.9661	0.9501	0.0160
Ethics	0.9754	0.9338	0.0416	Drama	0.9554	0.9389	0.0165
Music	0.9726	0.9310	0.0416	Cancer	0.9933	0.9764	0.0169
Imports	0.9830	0.9421	0.0409	Pension	0.9658	0.9482	0.0176
Water	0.9656	0.9249	0.0407	Marriage	0.9634	0.9458	0.0176
Flood	0.9374	0.8968	0.0406	Medicaid	0.9774	0.9595	0.0179
Hunting	0.9505	0.9108	0.0397	Sport	0.9547	0.9367	0.0180
Toy	0.9855	0.9460	0.0395	Mosque	0.9761	0.9579	0.0182
Agriculture	0.9810	0.9415	0.0395	Church	0.9762	0.9576	0.0186
Party	0.9369	0.8975	0.0394	Homicide	0.9791	0.9603	0.0188
Health	0.9495	0.9103	0.0392	Currency	0.9630	0.9436	0.0194
Automotive	0.9855	0.9466	0.0389	Prison	0.9688	0.9492	0.0196
LGBTQ	0.9928	0.9540	0.0388	Divorce	0.9752	0.9553	0.0199
Transport	0.9764	0.9387	0.0377	Drought	0.9543	0.9341	0.0202
Transfer	0.9494	0.9118	0.0376	Democracy	0.9819	0.9616	0.0203
Architecture	0.9875	0.9504	0.0371	Pope	0.9815	0.9611	0.0204
Stocks	0.9643	0.9277	0.0366	Celebrity	0.9573	0.9367	0.0206
Anniversary	0.9421	0.9056	0.0365	Epidemic	0.9393	0.9187	0.0206
University	0.9632	0.9267	0.0365	Inflation	0.9763	0.9556	0.0207
Employee	0.9380	0.9015	0.0365	Defence	0.9338	0.9131	0.0207
Fishing	0.9822	0.9457	0.0365	Hurricane	0.9687	0.9479	0.0208
Exports	0.9826	0.9462	0.0364	Loans	0.9752	0.9543	0.0209
Fiction	0.9607	0.9244	0.0363	Voting	0.9715	0.9506	0.0209
Illness	0.9515	0.9152	0.0363	Students	0.9598	0.9387	0.0211
Privacy	0.9893	0.9531	0.0362	Vaccines	0.9863	0.9652	0.0211
Employment	0.9533	0.9173	0.0360	Court	0.9226	0.9015	0.0211
Environment	0.9597	0.9241	0.0356	Pandemic	0.9273	0.9058	0.0215
Therapy	0.9736	0.9381	0.0355	Earthquake	0.9875	0.9659	0.0216
Animal	0.9828	0.9475	0.0353	Dance	0.9779	0.9558	0.0221
Children	0.9680	0.9329	0.0351	Bankruptcy	0.9364	0.9137	0.0227
Volleyball	0.9872	0.9523	0.0349	Tobacco	0.9847	0.9619	0.0228
Medicare	0.9700	0.9356	0.0344	Suicide	0.9754	0.9524	0.0230
Beverage	0.9872	0.9531	0.0341	Constitution	0.9633	0.9403	0.0230
Justice	0.9507	0.9168	0.0339	Disaster	0.9238	0.9005	0.0233
Fire	0.9697	0.9358	0.0339	Halloween	0.9913	0.9678	0.0235
Prices	0.9594	0.9258	0.0336	Teachers	0.9843	0.9607	0.0236
Easter	0.9810	0.9476	0.0334	Police	0.9649	0.9411	0.0238
Racism	0.9902	0.9570	0.0332	Media	0.9158	0.8919	0.0239
Tourism	0.9777	0.9446	0.0331	Petrol	0.9651	0.9412	0.0239
Trend	0.9216	0.8890	0.0326	Politics	0.9517	0.9269	0.0248
Farms	0.9734	0.9409	0.0325	Surgery	0.9787	0.9539	0.0248
Adoption	0.8417	0.8094	0.0323	Theatre	0.9776	0.9526	0.0250
Family	0.9572	0.9249	0.0323	Labour	0.9582	0.9330	0.0252
Abortion	0.9920	0.9598	0.0322	Welfare	0.9096	0.8832	0.0264
Terrorism	0.9791	0.9470	0.0321	Unemployment	0.9843	0.9577	0.0266
Culture	0.9476	0.9155	0.0321	School	0.9542	0.9276	0.0266
Gender	0.9776	0.9458	0.0318	College	0.9454	0.9188	0.0266
Plant	0.928	0.8964	0.0316	Holiday	0.9381	0.9112	0.0269
Rivers	0.9815	0.9499	0.0316	Poverty	0.9763	0.9493	0.0270
Game	0.9472	0.9157	0.0315	Economy	0.9648	0.9374	0.0274
Advertising	0.9667	0.9352	0.0315	Radio	0.9736	0.9461	0.0275
Sailing	0.9538	0.9224	0.0314	Wedding	0.9759	0.9483	0.0276
Education	0.9636	0.9323	0.0313	Fashion	0.9655	0.9379	0.0276
Securities	0.9762	0.9451	0.0311	Painting	0.9829	0.9552	0.0277
Logistics	0.9705	0.9397	0.0308	Consumers	0.9621	0.9343	0.0278
Ceremony	0.9333	0.9027	0.0306	Mortgage	0.9787	0.9508	0.0279
Insurance	0.9834	0.9529	0.0305	Government	0.9318	0.9033	0.0285
Bar	0.9553	0.9250	0.0303	Immigration	0.9900	0.9613	0.0287
Cafe	0.9852	0.9549	0.0303	Restaurant	0.9821	0.9533	0.0288

We also used the non-parametric Wilcoxon statistical test [48] to compare SVAE, and EOC-SVM methods considering the *F*_1_ values. Our SVAE outperforms EOC-SVM with a 95% confidence level.

**Fig 5:**
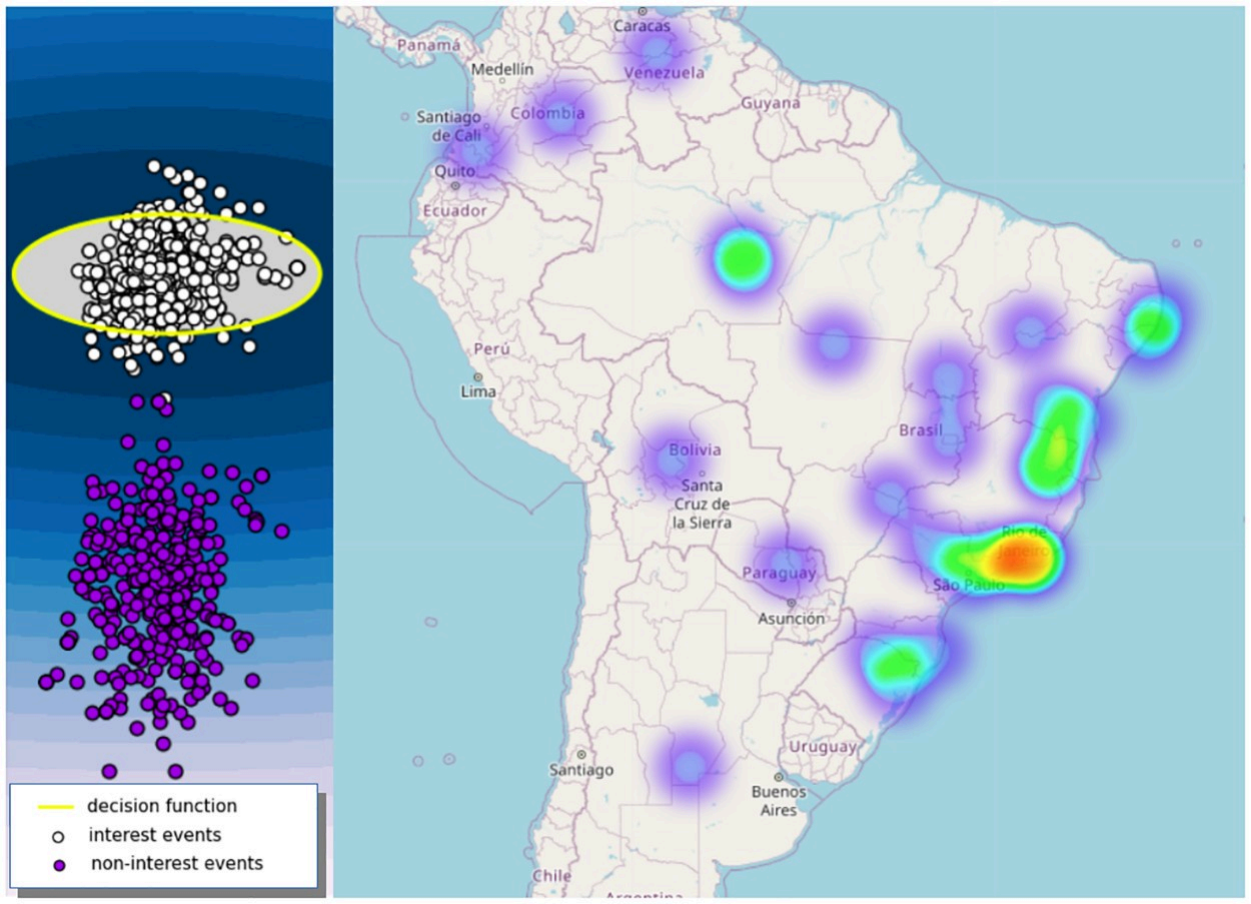
Sensor map about Brazilian Covid-19 events. Base map and data from OpenStreetMap and OpenStreetMap Foundation.

Besides the better classification performance of SVAE, our method also has the advantage of generating two-dimensional sensor maps that preserve semantic and spatio-temporal features. Fig 5 illustrates a sensor map about Brazilian Covid-19 events, particularly events that describe government actions on the pandemic. Note that the events belonging to the class of interest are allocated close to forming a high-density region. Then, OCSVM can obtain a better decision function to sense new events. We show a heatmap formed by the latitudes and longitudes coordinates extracted from the interest events to illustrate that sensors exploratory analysis can easily incorporate into the learning to sense tasks using our proposed SVAE. Note that our SVAE can be used as a predictive model. Furthermore, new collected events can be allocated incrementally on the sensor map and classified as belonging to the class of interest according to the learned decision function.

In order to show how much our proposal preserves geographic information, we carried out two experiments. In the first, we compared the correlation between the latitude and longitude information of events and the BERT‘s embeddings. We built a separate dataset with 1000 events about Coronavirus, using the same procedure as the 183 datasets collected for the experimental evaluation. In addition, we also collected the latitudes and longitudes of the events (spatial information). First, we construct the distance matrix *M*_*GEO*_ between pairs of events considering geographic information of latitude and longitude by using the Haversine distance. Second, we constructed the distance matrix *M*_*BERT*_ considering the representations generated by BERT, by using Euclidean distance. We then calculate the Spearman Rank correlation [49] *corr*(*M*_*GEO*_, *M*_*BERT*_) between 60 observations (event pairs) drawn randomly from each matrix. We defined 60 observations as this is the highest pre-calculated value to apply a Spearman Rank Correlation statistical significance test available in [50].

**Fig 6:**
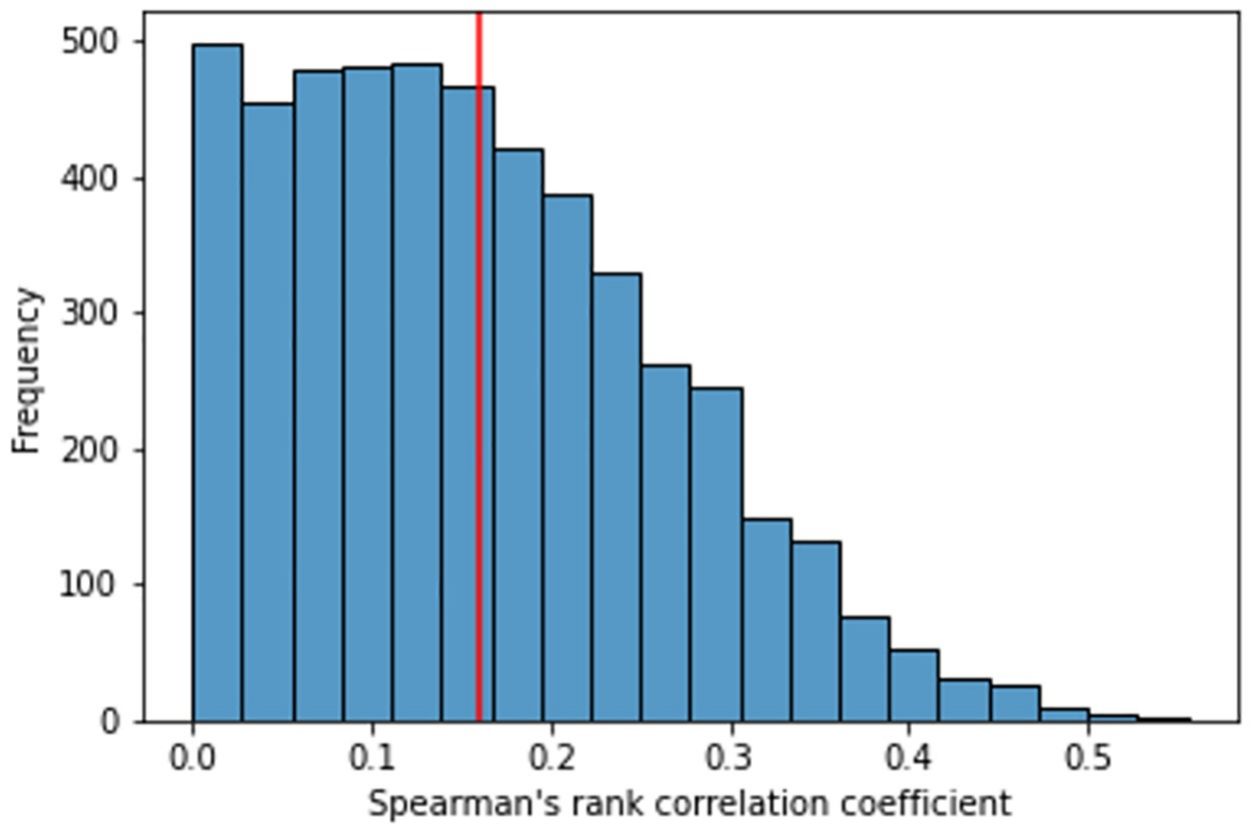
Histogram of frequency of Spearman correlation values between pairs of events considering *M*_*GEO*_ and *M*_*BERT*_.

Fig 6 shows the histogram with correlation values between BERT and original event geographical features in a simulation involving 5000 runs. The x-axis indicates the Spearman correlation value, and the y-axis indicates the frequency at which a correlation range was obtained. The red line indicates a threshold of statistical significance for the spearman correlation involving a sample size of 60 observations. From this threshold onwards, the correlations are significant with a 90% confidence level. We found that BERT is able to significantly capture geographic information in approximately 22% of runs.

**Fig 7:**
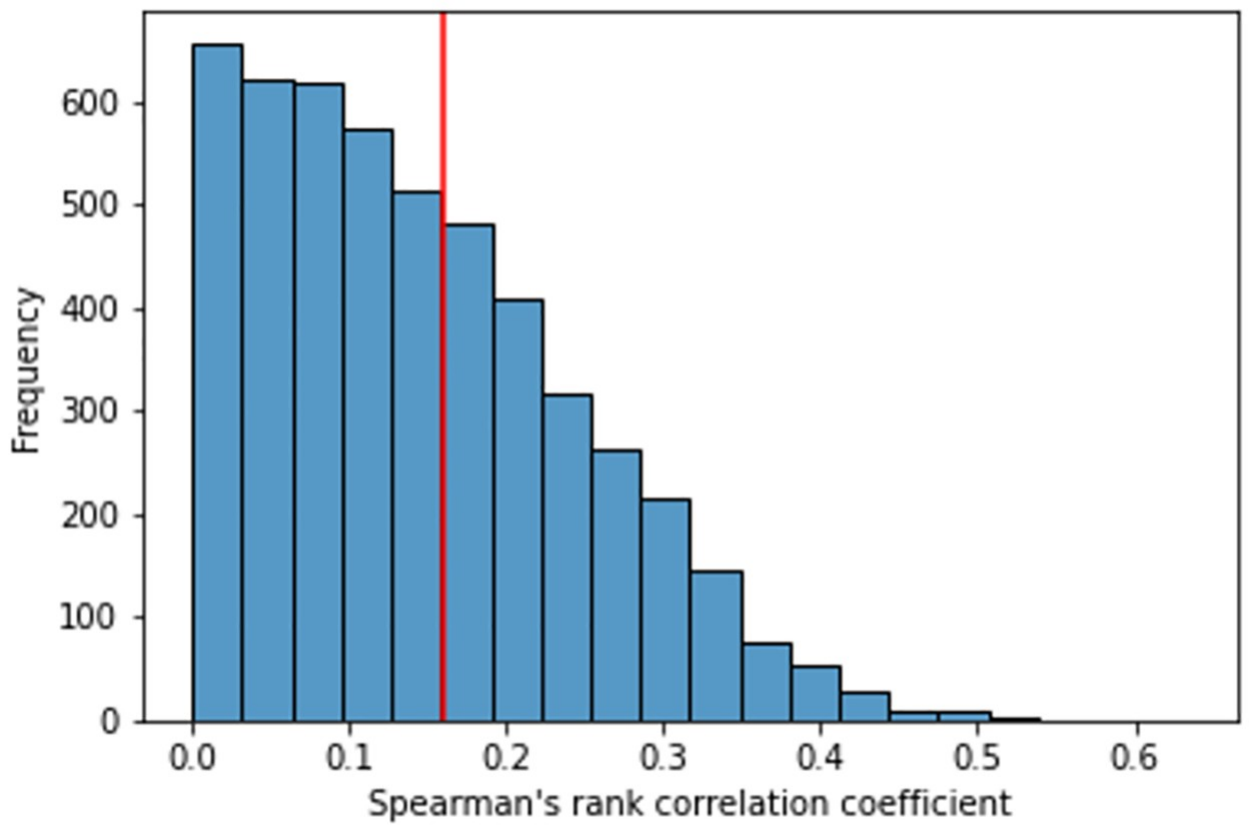
Histogram of frequency of Spearman correlation values between pairs of events *M*_*GEO*_ and *M*_*SVAE*_.

In the second experiment, we carried out the same process, however, we compared the correlation between the latitude and longitude information of events and the embeddings obtained by the SVAE (*corr*(*M*_*GEO*_, *M*_*SVAE*_)). Fig 7 shows the histogram with correlation values between SVAE and original event geographical features in a simulation involving 5000 runs. We found that SVAE is able to significantly preserves geographic information in approximately 20% of runs. We argue that this result is promising since: (i) coding from 512 dimensions to 2, the SVAE preserves the spatio-temporal information in 91% (20% of 22%); and (ii) the two simple features obtained by SVAE for sensor maps must preserve both textual content and geographic information. Furthermore, we train SVAE only with information from language models, in which geographic information can be implicitly presented in natural language such as the experiment presented previously.

## Concluding remarks

In this paper, we introduce the learning to sense tasks, in which one-class classification methods for texts are used to identify events of interest. We discussed the limitations of using existing methods for learning to sense, such as the lack of semantics and conceptual differences of these models for classical sensing tasks. In practice, learning to sense tries to emulate human reasoning to monitor and interpret events of interest for decision-making processes.

We propose a method called SVAE (Semantic Variational Autoencoder) to learn two-dimensional sensor maps capable of extracting non-linear features and preserving properties extracted from neural language models, in which events are represented via embeddings. Also, our SVAE tends to preserve existing spatio-temporal features of the events. Our experimental evaluation indicated that the proposed SVAE outperforms a robust one-class classification method based on BERT embeddings. Moreover, our SVAE has desirable characteristics for event analysis, such as visualization of the classifiers’ function decision and facilitating the visualization of the geographic impact of the events of interest through heat maps.

Directions for future work involve training specific neural language models for large collections of events to highlight spatio-temporal features in contextual word embeddings and a study about the impact of the bottleneck sizes in the learning to sense performance. Deep autoencoders are also promising, particularly combining convolutional neural networks and variational autoencoders to obtain better sensor maps. Finally, future work involves the creation of a multimodal version of SVAE to explore the combination of BERT embeddings and geolocations (latitude and longitude).
